# The metastasis suppressor Nm23 as a modulator of Ras/ERK signaling

**DOI:** 10.1186/1750-2187-9-4

**Published:** 2014-05-12

**Authors:** Krisztina Takács-Vellai

**Affiliations:** 1 Department of Biological Anthropology, Eötvös Loránd University, Pázmány Péter stny. 1/C, H-1117 Budapest, Hungary

**Keywords:** Metastasis inhibitor, Nm23, Nucleoside-diphosphate kinase (NDPK), Kinase, Ras/ERK signaling, Kinase suppressor of Ras (KSR), Scaffold

## Abstract

NM23-H1 (also known as NME1) was the first identified metastasis suppressor, which displays a nucleoside diphosphate kinase (NDPK) and histidine protein kinase activity. NDPKs are linked to many processes, such as cell migration, proliferation, differentiation, but the exact mechanism whereby NM23-H1 inhibits the metastatic potential of cancer cells remains elusive. However, some recent data suggest that NM23-H1 may exert its anti-metastatic effect by blocking Ras/ERK signaling. In mammalian cell lines NDPK-mediated attenuation of Ras/ERK signaling occurs through phosphorylation (thus inactivation) of KSR (kinase suppressor of Ras) scaffolds. In this review I summarize our knowledge about KSR’s function and its regulation in mammals and in *C. elegans*. Genetic studies in the nematode contributed substantially to our understanding of the function and regulation of the Ras pathway (i.e. KSR’s discovery is also linked to the nematode). Components of the RTK/Ras/ERK pathway seem to be highly conserved between mammals and worms. NDK-1, the worm homolog of NM23-H1 affects Ras/MAPK signaling at the level of KSRs, and a functional interaction between NDK-1/NDPK and KSRs was first demonstrated in the worm *in vivo*. However, NDK-1 is a factor, which is necessary for proper MAPK activation, thus it activates rather than suppresses Ras/MAPK signaling in the worm. The contradiction between results in mammalian cell lines and in the worm regarding NDPKs’ effect exerted on the outcome of Ras signaling might be resolved, if we better understand the function, structure and regulation of KSR scaffolds.

## Review

### Introduction

*nm23-H1*, also known as *nme1*, was the first identified metastasis suppressor gene [1]. The reverse correlation between NM23 expression and metastatic potential had been reported many times using cell lines and xenograft models (reviewed in [2]). The metastasis inhibitor effect was also demonstrated using mouse models: double transgenic mice derived from a cross of *nm23-M1* knockout mice with a mouse strain prone to hepatocellular carcinoma show a higher incidence of lung metastases [3]. Members of the *nme* gene family encode nucleoside diphosphate kinases (NDPK) [4]. In recent years NDPKs have been ascribed numerous roles in development that are inconsistent with a simple housekeeping role of balancing pools of nucleoside diphosphates and triphosphates. Recent data suggest that members of the *nm23* family display multiple functions in diverse biological processes such as signal transduction, growth control, differentiation, cell migration, cancer promotion [5-7]. Although the exact molecular process by which NM23 inhibits the metastatic potential of cancer cells is unknown, some molecular mechanisms have been proposed to explain its anti-metastatic activity. NM23 silencing in hepatoma and colon carcinoma cell lines resulted in upregulation of the membrane associated matrix metalloproteinase (MT1-MMP), increased Rac1 signaling and activation of several pro-invasive signaling pathways such as MAPK (mitogen activated protein kinase)/SAPK (stress-activated protein kinases), Akt [8]. Elevated Rac1 level upon NM23 knockdown suggests that NM23 inhibits Rac1 activation. Although the exact mechanism is unknown, NM23-H1 was shown to interact with factors involved in Rac1 activation, such as the small GTPase Arf6 or the nucleotide exchange factor Tiam1 [9,10]. ARF6 by recruiting NM23-H1 was reported to facilitate dynamin-mediated endocytosis during adherens junctions disassembly and NM23-H1 was suggested to provide a source of GTP for ARF6 [9]. The highly specific functional relationship between dynamin and the NM23 homolog AWD in endocytosis was demonstrated by genetic studies in *Drosophila*[11]. AWD functions as a negative regulator of cell migration in tracheal and border cells by downregulating receptor levels on the cell surface through dynamin-mediated endocytosis [12,13], thus it suppresses cell motility through receptor internalization. Some studies suggest that NM23-H1/H2 may exert its anti-metastatic effect by blocking Ras/ERK (extracellular signal-regulated kinase) signaling [14-17]. In this review I summarize how NM23-H1 inhibits the Ras/ERK cascade in human cell lines, and examine how the NM23 homolog NDK-1 modifies the outcome of Ras signaling in the model organism *Caenorhabditis elegans*[18].

### The RTK/Ras/ERK pathway: its human relevance and significance during nematode development

RTK (receptor tyrosine kinase)/Ras/ERK signaling plays a critical role in a wide range of biological processes such as cell proliferation and differentiation, cell migration and survival, cellular metabolism (reviewed in [19]). Mutations affecting RTK/Ras/ERK signaling cause human syndromes called rasopathies, including Noonan syndrome, Costello syndrome [20] and tumorigenesis [21].

RTKs often signal to the Ras/ERK cascade via GRB-2 (growth factor receptor-bound protein 2) and the Guanine Nucleotide Exchange Factor (GEF) Sos (son of sevenless) to activate the small GTPase Ras. Subsequently Ras-GTP binds to Raf and promotes its association with the plasma membrane and/or endomembranes [22], where Raf activation occurs. The scaffold protein KSR (kinase suppressor of Ras) helps in Raf activation, but also contributes to the sequential phosphorylation steps of the MAPK cascade by bringing together MAPK components, Raf, MEK and ERK/MAPK. Sequential phosphorylation within the MAPK modul leads to ERK activation, which often results in changes in gene expression [23].

### Canonical RTK/Ras/ERK pathway plays multiple biological roles during *C. elegans* development

Studies in the nematode *Caenorhabditis elegans* contributed substantially to our knowledge about the mechanism and biological function of the canonical RTK/Ras/ERK pathway. Although nematode RTK/Ras/ERK components at the level of protein-protein interactions or protein modifications have not been so deeply investigated as their human counterparts, the canonical pathway seems to be highly conserved between the nematode and mammals (reviewed in [24]).

Variations of RTK/Ras/ERK signaling in given cell types, tissues or developmental events use distinct ligands and RTKs, which transmit the signal to the Ras/ERK cytoplasmic signaling modul. ERK activation in these processes results in regulation of different target genes leading to specification of different cell types and contributing to development of different organs. RTK/Ras/ERK signaling using the ligand LIN-3/EGF and its receptor LET-23/EGFR initiates vulval induction. The EGFR/Ras/ERK pathway plays an essential, inductive role during development of the vulva, the hermaphrodite reproductive organ [25]. Mutations causing reduced Ras signaling (for example loss-of-function or hypomorph alleles of *let-23/EGFR* or *let-60/Ras*) result in Vulvaless phenotype, whereas increased Ras signaling (for example caused by gain-of-function alleles of *let-23, let-60*) leads to a Multivulva phenotype (reviewed in [24]).

### KSR (kinase suppressor of Ras), a well known scaffold protein of the Ras/MAPK cascade

*ksr* (kinase suppressor of Ras) genes were initially identified in *C. elegans* and *Drosophila melanogaster* as positive regulators of Ras/MAPK signaling [26-28].

The *Drosophila* genome encodes only a single *ksr* gene which is essential for viability in the fly [28]. In the worm, mutations in *ksr-1* were isolated in genetic screens searching for suppressors of the Muv (multivulva) phenotype caused by a gain-of-function allele of *let-60/Ras*[26,27]. In contrast to its fly counterpart, *ksr-1* is not an essential gene as *ksr-1* null mutants are viable and do not show severe defects in development. However, subsequently a second *ksr* ortholog was identified in the nematode genome, *ksr-2*[29]. In the germline Ras/MAPK signaling driven by an unknown ligand mediates progression of germ cells through the meiotic pachytene stage [30]. *ksr-2* loss-of-function mutants display a sterile gonad with germ cells arrested at pachytene, similar to null mutants of other MAPK cascade genes [29,31].

Inactivation of both worm *ksr* paralogs, *ksr-1* and *ksr-2* resulted in “rod-like” L1 lethality and Vulvaless phenotype, reminiscent of strong *let-60/Ras* mutant phenotypes, and revealed that the two *ksr* paralogs function redundantly in many processes during worm development and play a key role in Ras-mediated signaling events [29].

The mouse genome contains two *KSR* paralogs, KSR1 and KSR2. KSR1 knockout mice are viable and do not display major developmental defects [32,33], suggesting large degree of functional redundancy between the different isoforms in this organism as well. However in KSR1−/− mice, as a consequence of decreased MAPK signaling T-cell activation is impaired [34], these animals have enlarged adipocytes [35] and are slightly glucose intolerant [32]. Glucose homeostasis is regulated by the MARK2 kinase, which phosphorylates and inactivates KSR1, thus leading to dampened ERK signaling [32]. KSR2−/− mice were found obese and also glucose intolerant [36]. Obesity in KSR2−/− mice is linked to AMPK dependent glucose uptake and fatty acid oxidation, as KSR2 interacts with AMPK and modulates its activity [37]. Mutations in KSR-2 were recently described to be associated with obesity and insulin resistance in human [38]. It is important to note that mouse KSR1−/−; KSR2−/− double mutants have not yet been described.

KSRs are known as scaffold proteins which coordinate the assembly of membrane-localized Raf/MEK/ERK complexes [39-41]. KSR1 is able to bind all three kinases of the MAPK pathway: it associates constitutively with MEK, but interaction with C-Raf and ERK1/2 occurs only upon growth factor stimulation [39,42]. Thus, upon Ras activation KSR brings MEK to the plasma membrane in close proximity to the Raf kinase. In principle, KSR provides a scaffold, which facilitates the phosphorylation steps in the MAPK cascade in order to execute signal transduction downstream of Ras.

**Figure 1:**
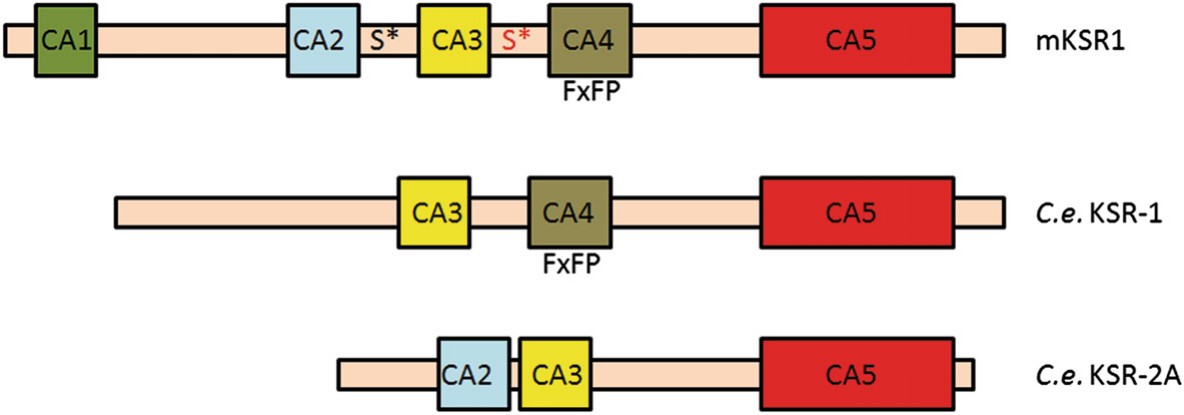
**Domain organization of the worm KSR proteins related to murine KSR1.** KSR proteins consist of five conserved areas (CA): CA1 (KSR-specific domain), CA2 (proline-rich region), CA3 (cysteine-rich region), CA4 (serine/threonine-rich region), CA5 (kinase-like domain). *C. elegans* (*C.e.*) KSR-1 and KSR-2A lack the CA1 domain. Worm KSR-1 lacks the CA2 domain, whereas in KSR-2A the CA4 domain with the FXFP motif (ERK/MAPK docking site) is absent. 14-3-3 binding sites Ser297 (S*) and Ser392 (S* marked red) located on either side of the CA3 domain in the mouse protein (mKSR1) are absent in the worm proteins.

The domain structure of KSR proteins is similar to that of Raf family members indicating their possible common origin (reviewed in [41]). KSR proteins consist of five conserved regions, termed CA1–CA5 (summarized in Figure 1). The N-terminal CA1 domain is unique for KSRs, this domain was shown to bind Raf in the *Drosophila* KSR homolog (reviewed in [43]). The CA2 domain contains a proline-rich region, whereas the CA3 domain possess a cysteine-rich region. The CA3 domain is able to bind lipids, it ensures membrane anchorage of KSR upon Ras activation, thus this domain regulates the cellular localization of KSR [44]. The CA4 domain contains a serine/threonine-rich sequence and conserved sites (FXFP, Figure 1) for ERK docking [41,45]. Finally, the CA5 domain is a C-terminal kinase-like domain, which is similar to the kinase domain of Raf proteins and seems to be important for MEK binding [46]. KSR1 is considered to be a pseudokinase that lacks catalytic activity because of the absence of an invariant Lys residue in subdomain I, which is important for orienting ATP in Raf. This critical Lys is exchanged for Arg in human KSR1, but we note that *Drosophila* KSR and *C. elegans* KSR-1 (but not KSR-2) contain Lys in the conserved position. Together, to date still debates exist about KSR’s catalytic activity.

Recent studies elaborated the mechanism of Raf activation and highlighted KSR’s function in this process. In mammalian cells there are three isoforms of Raf: A-Raf, B-Raf and C-Raf. Recent data suggest that dimerization plays a key role in Raf activation. Hu and colleagues [47] showed that Raf dimers are asymmetric, they are composed of an activator kinase (for instance B-Raf) and a receiver kinase (such as C-Raf). The activator kinase requires no kinase activity but requires N-terminal (NtA: N-terminal acidic motif) phosphorylation to allosterically stimulate *cis*-autophosphorylation and transactivation of the partner receiver kinase. KSR can be also involved in dimerization and Raf activation. If the NtA is phosphorylated on KSR1 or C-Raf, both can function as activator kinases of their partners C-Raf or B-Raf. Moreover, Brennan and co-workers showed that B-Raf dimerization with KSR2 could allosterically stimulate the kinase activity of KSR2 towards MEK [48], however it is not known yet whether this transactivation event is mediated by the NtA of B-Raf. Studies on B-Raf inhibitor drugs led to the observation that unphosphorylated KSR1, however, is able to block B-Raf activation by constitutively dimerizing with B-Raf [49]. Thus, KSR represents a critical factor in modulation of the MAPK pathway.

Sequence comparison of the worm KSR proteins reveals that *C. elegans* KSR-1 and KSR-2 share 26% and 31% overall sequence identity with murine KSR1 [18,29]. Genetic analyses show evidences that worm KSR-1 and KSR-2 are both functional KSR family members [26,27,29], however differences can be seen in their domain structure compared with murine KSR1, the best characterized mammalian KSR (Figure 1). The CA1 domain is missing in both *C. elegans* KSR homologs, in addition, worm KSR-1 lacks the CA2 domain, whereas in KSR-2 the CA4 domain with the ERK docking site is absent (Figure 1).

### Regulation of KSR: NM23/NDPK is a factor, which is able to phosphorylate KSR1 on Ser392

Upon Ras activation, KSR1 is localized to the plasma membrane, but in unstimulated cells KSR can be found in the cytoplasm. KSR localization is known to be regulated by protein interactions, through phosphorylation and dephosphorylation steps. In quiescent cells KSR is phosphorylated on Ser297 and Ser392 residues (residues are numbered according to the mouse KSR1 sequence), 14-3-3 protein binds to these phosphorylated sites and keeps KSR in the cytoplasm [42]. 14-3-3 protein is able to mask the CA3 domain of KSR together with IMP (impedes of mitogenic signal propagation), an E3 ubiquitin ligase, which also binds to the CA3 region of KSR to inhibit plasma membrane targeting [50].

Growth factor stimulation and Ras activation lead to multiple changes in the cytosolic KSR complex: Ras binds to IMP, which is degraded subsequently; upon growth factor stimulus, the Ser/Thr protein phosphatase PP2A dephosphorylates KSR1 on the Ser392 residue [51]. As a result, KSR is released of the inhibition exerted by 14-3-3 and translocates to the plasma membrane to promote MAPK pathway activation.

**Figure 2:**
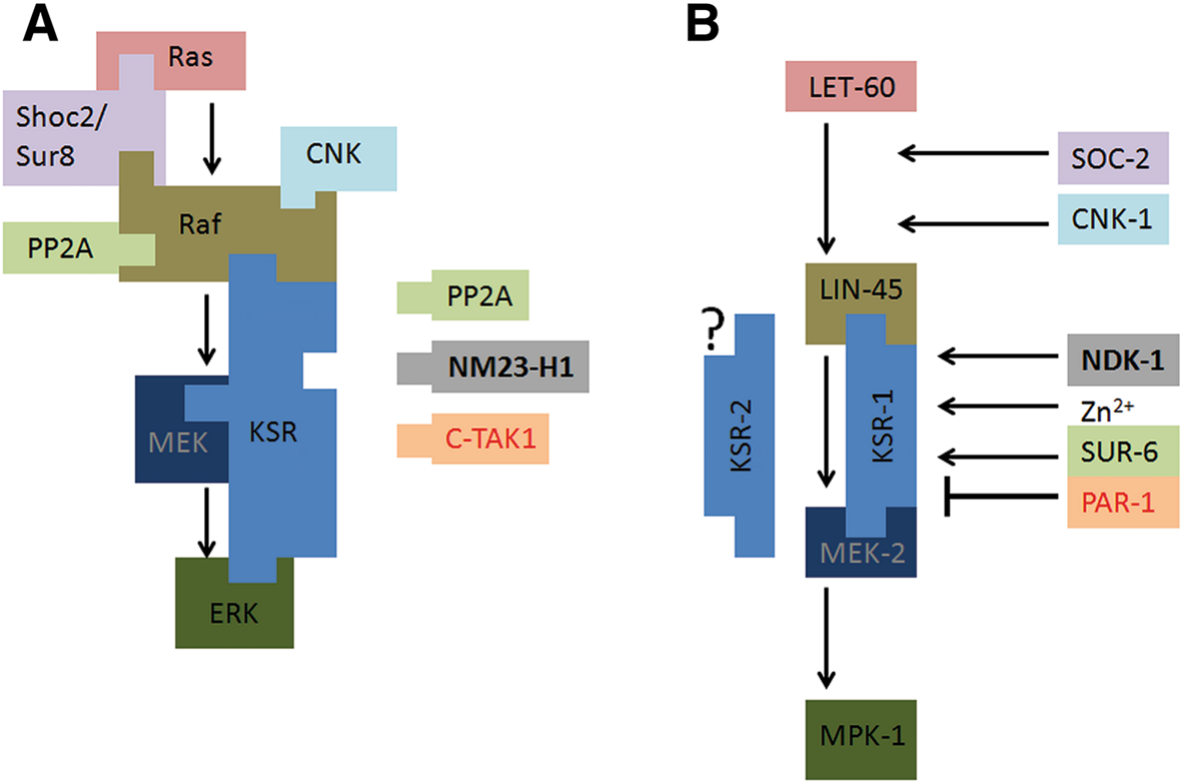
**Scaffolds of the Raf/MEK/ERK cascade and regulation of KSR in mammals and in the nematode.** SOC-2/SUR-8, CNK/CNK-1 and KSR are scaffolds of the Raf/MEK/ERK cascade. SOC-2/SUR-8 and CNK/CNK-1 are thought to facilitate Raf activation [52,53]. KSR assembles Raf/MEK/ERK complexes and functions downstream of Raf. **A)** Protein interactions in mammals [22]. C-TAK1 and Nm23-H1 phosphorylate Ser392 of mammalian KSR, while PP2A is able to dephosphorylate the same residue. **B)** Genetic interactions in *C. elegans*. PAR-1 inhibits, while SUR-6/PP2A activates KSR-1. NDK-1 activates Ras signaling at the level of KSRs. The molecular mechanisms are not known. The regulation of KSR-2 remains to be determined. The mammalian proteins and their nematode homologs are labeled by the same color.

Phosphorylation and dephosphorylation events occuring on Ser392 residue are critical in respect of KSR regulation (Figure 2A). As mentioned above, dephosphorylation of Ser392 by PP2A results in KSR activation. In contrast, phosphorylation of this residue for example by the Cdc25C-associated kinase 1 (C-TAK1) mediates the binding of KSR to 14-3-3 proteins, driving cytoplasmic sequestration of KSR (i.e. inactivation of KSR) in unstimulated cells [42] (Figure 2A).

C-TAK-1 is not the only kinase phosphorylating KSR on Ser392 residue. The best known NDPK, human NM23-H1, which is characterized by histidine protein kinase activity [54], was also found to phosphorylate one of the 14-3-3 binding sites of KSR1, Ser392 through a histidine intermediate [14] (Figure 2A). Overexpression of NM23-H1 in MDA-MB-435 breast carcinoma cells resulted in decreased activated MAPK (pMAPK) levels without affecting the level of total MAPK, suggesting that NM23-H1 inhibits Ras/MAPK signaling through KSR phosphorylation and inactivation [14]. In MDA-MB-435 cells transfected by NM23-H1 increased co-immunoprecipitation of Hsp90 with KSR1 was observed [15]. Hsp90 is a binding partner of KSR, which might promote the proteasome-mediated degradation of KSR, thus attenuating ERK signaling.

Modulators of KSR activity were also identified in *C. elegans*. As EGFR/Ras/ERK activity is indispensable for vulval induction, differences in the level of MAPK signaling can be measured by scoring the formation of a reduced or enhanced number of vulval structures. Thus, the vulval induction system provides a unique opportunity to identify regulators of the Ras/ERK cascade. Genetic epistasis analyses demonstrated that the C-TAK1 homolog PAR-1 kinase and the worm counterpart of PP2A phosphatase, SUR-6/PP2A act downstream of LIN-45/Raf, at the level of KSR-1 [52,55]. PAR-1 regulated KSR-1 activity negatively, whereas SUR-6/PP2A modified the effect of KSR-1 positively, similar to data derived from human cell lines [52,55] (Figure 2B). However, as 14-3-3 binding sites Ser297 and Ser392 are not present in the worm KSR-1 protein (Figure 1), the exact mechanism of action whereby PAR-1 and SUR-6/PP2A modify KSR activity remains elusive.

### NDK-1, the *C. elegans* NM23-H1/2 homolog is required for full activation of Ras/ERK signaling

Our group has recently characterized *ndk-1*, the *C. elegans* ortholog of human *nm23-H1/H2*[18]. NDK-1 shows 65% sequence identity and 85% overall similarity to NM23-H1 and NM23-H2. 50% of *ndk-1* loss-of-function mutants die as embryos, the remainder develop into sterile adults, which display a protruding vulva (Pvl) phenotype.

**Figure 3:**
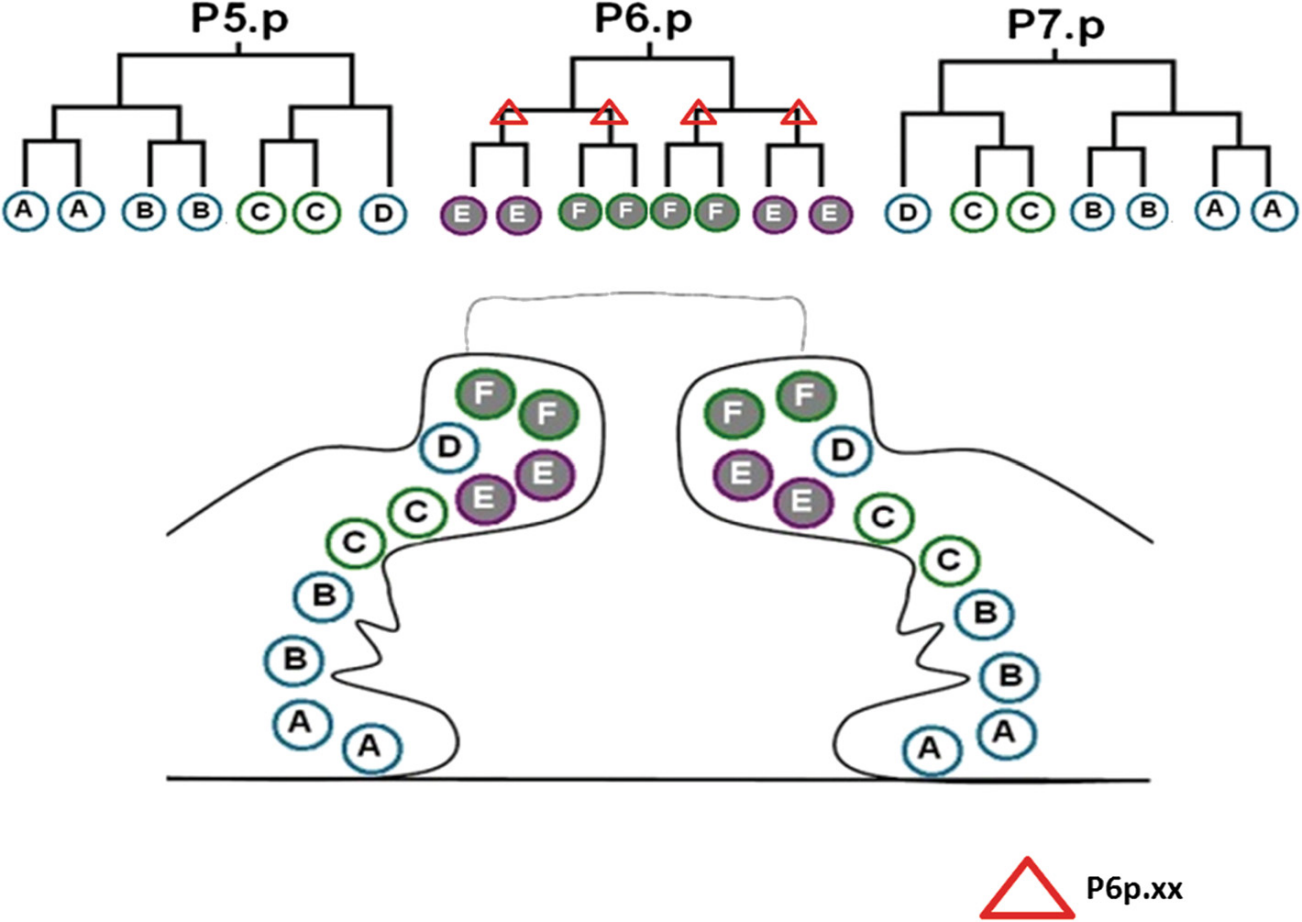
**EGL-17/FGF, a known MAPK target shows reduced expression in *ndk-1(−) *mutant background.** The vulva of wild-type hermaphrodites develops from a subset of epidermal blast cells, called vulval precursor cells (VPCs). P(5–7).p VPCs undergo three rounds of cell division and give rise to 22 vulval cells. The vulva at mid-L4 larval stage is composed of 22 cells, which can be grouped into seven cell types (vulA, B1, B2, C, D, E and F). These cells are the great-granddaughters of P5.p-P7.p VPCs (grey filled circles: primary lineages; white circles: secondary lineages). EGL-17/FGF is a known MAPK target, which is expressed in all daughters (P6p.x) and granddaughters (P6p.xx) of P6.p in the L3 stage in wild-type animals. EGL-17/FGF expression was reduced in P6p.xx cells (red triangle) in *ndk-1(−)* mutants suggesting that Ras/MAPK signaling is inhibited in the absence of NDK-1 [18].

The vulva of the *C. elegans* hermaphrodite develops from a subset of epidermal blast cells, called vulval precursor cells (VPCs). Briefly, at the early L3 larval stage three VPCs, P5.p, P6.p and P7.p receive an inductive signal conferred by an EGF ligand derived from a specific cell of the somatic gonad, the anchor cell (Figure 3). EGF ligand activates the EGFR/Ras/ERK pathway in P(5–7).p cells (reviewed in [25]). P6.p, the VPC closest to the anchor cell, adopts the primary vulval fate, while the neighboring VPCs, P5.p and P7.p adopt the secondary vulval fate as a result of lateral signaling mediated by the LIN-12/Notch pathway. The induced VPCs undergo three rounds of cell divisions and finally result in 22 vulval cells in the L4 larval stage. P6.p descendants are primary lineages, whereas descendants of P5.p and P7.p are secondary vulval cells (Figure 3). These 22 cells undergo cell migration and fusion events to eventually form the adult vulval structure (reviewed in [25]). In Pvl mutants a single protrusion is formed, which is caused by eversion of the vulval tissue [56].

By analyzing the vulva phenotype, we demonstrated that the number of vulva cells in *ndk-1(−)* mutants is decreased compared with the 22 cells observed in the wild type. The Pvl phenotype might occur because of misspecification of vulval cell fates, both primary and secondary vulval cell fates are affected in the mutants [18]. Next we assessed how NDK-1 modifies Ras/MAPK signaling. *egl-17*/*FGF* is a known MAPK target, which is expressed in primary vulval lineages during L3 larval stage [57]. We observed a reduced *egl-17*/*FGF* expression in P6.p granddaughter cells in the developing vulval tissue of *ndk-1* mutants (Figure 3). Epistasis analysis performed with *ndk-1* and Ras pathway mutants demonstrated that *ndk-1* acts downstream of *lin- 45/Raf* and upstream of *mek-2/MEK* and *mpk-1/MAPK* to affect the formation of the vulva tissue. In addition, we measured a strongly decreased level of activated MAPK in somatic tissues of *ndk-1* knockouts, whereas total MAPK levels were comparable with wild-type animals [18]. Together, these results show that NDK-1 is necessary for proper MAPK activation in somatic tissues of the worm. Since NDK-1 and the KSR paralogs function at the same level in the Ras/ERK cascade and they all contribute to MAPK activation, we generated *ndk-1(−);ksr-1(−)* and *ndk-1(−)ksr-2(−)* double knockouts in order to test their potential genetic interactions. In the double mutants enhancement of single-mutant phenotypes was observed, in particular in case of *ndk-1(−)ksr-2(−)*, where embryonic lethality of homozygous *ndk-1(−)* mutants (50%) becomes fully penetrant. The complete lethality of *ndk-1(−)ksr-2(−)* double mutants suggest that NDK-1 and KSR-2 display essential functions during *C. elegans* embryogenesis. *In vitro* pulldown experiments showed that NDK-1 binds to worm KSR-2 and murine KSR1.

## Conclusions

In the human breast carcinoma cell line MDA-MB435 and in HEK293 cells NM23-H1 has been shown to phosphorylate KSR1 on Ser392 [14,15,17], and NM23-H1 overexpression in those cells resulted in reduced levels of MAPK signaling. Conversely, silencing of NM23-H1 in HepG2 hepatocellular carcinoma cells induced elevated phospho-ERK levels [8]. It was also suggested that increased levels of NM23-H2 block the ERK pathway [16]. Thus, NDPKs might attenuate Ras/ERK signaling through phosphorylation (thus inactivation) of KSR scaffolds.

Our group has recently characterized NDK-1, the *C. elegans* homolog of group I NDPKs, and we provided a compelling case that NDK-1 affects Ras/MAPK signaling at the level of KSRs, and first demonstrated a functional interaction between NDK-1/NDPK and KSRs *in vivo*[18]. However, genetic and biochemical data demonstrate reduced MAPK signaling in somatic tissues of *ndk-1* knockouts, therefore we conclude that NDK-1 is necessary for proper MAPK activation, thus NDK-1/NDPK exerts a stimulatory effect on Ras/MAPK signaling [18]. Since NDK-1 directly interacts with KSR-2 and with murine KSR1, we consider it likely that NDK-1 modifies the function of KSR scaffolds.

Contradictions between mammalian and *C. elegans* data regarding the effect of NDPKs on the outcome of Ras/ERK signaling could be resolved if we better understand the function and regulation of KSR scaffolds. Mouse KSR1 harbors 14-3-3 binding sites Ser297 and Ser392 that are involved in KSR scaffold regulation in mammals [42]. The implicated residues, however, are evolutionary not conserved in *C. elegans* KSR proteins (Figure 1) [29], suggesting that a 14-3-3 domain binding triggered attenuation may not be operating in the worm. Genetic data demonstrate that the C-TAK1 homolog PAR-1 kinase and SUR-6/PP2A phosphatase are involved in the regulation of worm KSR-1 similar to their human counterparts, but the exact KSR-1 residues where PAR-1 and SUR-6/PP2A act, are not known (Figure 2) [52,55]. Worm KSR-2 seems to be the most divergent member of the KSR family, as it lacks the CA1 region and the CA4 domain, which contains the ERK docking site (Figure 1) [29]. Therefore, it is possible that KSR-2 might function differently from other KSR proteins, and its regulation might involve other mechanisms. It is also important to consider that Raf activation might occur differently in the worm compared to mammals, as in *C. elegans* only B-Raf (LIN-45) is present but not C-Raf [24]. Novel studies suggest that dimerization involving KSR is crucial for Raf activation, in addition unphosphorylated KSR might exert an inhibitory effect on Raf activation [47-49]. Not only the phosphorylation status of KSR, but also its expression level might be important regarding the outcome of MAPK signaling, as early studies on overexpression of mammalian KSR pointed to a negative role for KSR in signaling, while later loss-of-function studies suggested a positive role (reviewed in [39]).

Together, further studies are necessary to dissect the molecular mechanism by which NDK-1 influences KSR activity. These studies could provide further data about regulation of scaffolds, as scaffolds are known to act not only as passive signaling platforms, but active surfaces, whose changes might significantly influence the outcome of signaling [58].

KSRs might also function differently in distinct organisms. In the worm KSR activity is indispensable for Ras signaling, as inactivation of both *ksr* paralogs by RNAi results in L1 lethality (e.g. strong *ras*-like phenotype) and absent levels of activated MAPK [29]. In contrast, in KSR-1 knockout mice the high molecular weight complexes containing KSR, MEK and ERK are not assembled, although the mutants are grossly normal [34]. These data suggest that mouse KSR1 is not strictly required for, but rather enhances MAPK signaling. However, to date there are no data available about the phenotype and activated MAPK levels of KSR1−/−;KSR2−/− double mutant mice.

Recently pioneering work has shown that in HEK293 cells RGS19 (regulator of G protein signaling 19) is able to suppress Ras signaling through upregulation of Nm23 expression and NM23-mediated phosphorylation of KSR [17]. KSR and Nm23 were also linked to G protein signaling [59,60]. RGS19 is classified as a member of the RZ/A subfamily of RGS proteins [61]. This subfamily is also represented in the worm by RGS-1 and RGS-2 although they lack an N-terminal cysteine string, which is present in human family members [62,63]. Further investigations are necessary to explore, whether the function of *C. elegans* RGS-1 and RGS-2 is related to the NDK-1/KSR interaction, and to see how transcriptional activation of *ndk-1/nm23* is regulated. Nevertheless, mutagenesis screens could be performed in *C. elegans* in order to identify upstream regulators of *ndk-1/nm23.*

## Competing interests

The author declares no competing interests.
